# Cochlear implant revision surgeries in children^☆^

**DOI:** 10.1016/j.bjorl.2018.01.003

**Published:** 2018-02-16

**Authors:** Maria Stella Arantes do Amaral, Ana Cláudia Mirândola B. Reis, Eduardo T. Massuda, Miguel Angelo Hyppolito

**Affiliations:** a Universidade de São Paulo (USP), Pós Graduação, Faculdade de Medicina de Ribeirão Preto, Hospital das Clínicas, Divisão de Otorrinolaringologia, Ribeirão Preto, SP, Brazil; b Universidade de São Paulo (USP), Faculdade de Medicina de Ribeirão Preto, Departamento de Oftalmologia, Otorrinolaringologia e Cirurgia de Cabeça e Pescoço, Ribeirão Preto, SP, Brazil

**Keywords:** Cochlear implant, Sensorineural hearing loss, Hearing loss, Implante coclear, Perda auditiva neurossensorial, Surdez

## Abstract

**Introduction:**

The surgery during which the cochlear implant internal device is implanted is not entirely free of risks and may produce problems that will require revision surgeries.

**Objective:**

To verify the indications for cochlear implantation revision surgery for the cochlear implant internal device, its effectiveness and its correlation with certain variables related to language and hearing.

**Methods:**

A retrospective study of patients under 18 years submitted to cochlear implant surgery from 2004 to 2015 in a public hospital in Brazil. Data collected were: age at the time of implantation, gender, etiology of the hearing loss, audiological and oral language characteristics of each patient before and after cochlear implant surgery and any need for surgical revision and the reason for it.

**Results:**

Two hundred and sixty-five surgeries were performed in 236 patients. Eight patients received a bilateral cochlear implant and 10 patients required revision surgery. Thirty-two surgeries were necessary for these 10 children (1 bilateral cochlear implant), of which 21 were revision surgeries. In 2 children, cochlear implant removal was necessary, without reimplantation, one with cochlear malformation due to incomplete partition type I and another due to trauma. With respect to the cause for revision surgery, of the 8 children who were successfully reimplanted, four had cochlear calcification following meningitis, one followed trauma, one exhibited a facial nerve malformation, one experienced a failure of the cochlear implant internal device and one revision surgery was necessary because the electrode was twisted.

**Conclusion:**

The incidence of the cochlear implant revision surgery was 4.23%. The period following the revision surgeries revealed an improvement in the subject's hearing and language performance, indicating that these surgeries are valid in most cases.

## Resumo

**Introdução:**

A cirurgia para a implantação do dispositivo interno do implante coclear não é inteiramente livre de riscos e pode apresentar problemas que exigem cirurgias revisionais.

**Objetivo:**

Verificar a incidência das indicações de cirurgias revisionais para a implantação do dispositivo interno do implante coclear, sua efetividade e correlacionar às variáveis relacionadas às categorias de linguagem e audição.

**Método:**

Estudo retrospectivo de pacientes menores de 18 anos submetidos à cirurgia de implante coclear de 2004 a 2015 em um hospital público no Brasil. Os dados coletados foram: idade no momento da implantação, sexo, etiologia da surdez, características audiológicas e da linguagem oral de cada paciente no pré e pós-operatório, necessidade de revisão cirúrgica e razão para ela.

**Resultados:**

Foram feitas 265 cirurgias em 236 pacientes. Oito pacientes foram submetidos ao implante coclear bilateral e 10 necessitaram de cirurgia de revisão. Foram necessárias 32 cirurgias para essas 10 crianças (um implante coclear bilateral), das quais 21 foram cirurgias de revisão. Em duas crianças, a remoção do implante coclear foi necessária, sem realização de reimplante; sendo que uma delas apresentava malformação coclear, por partição incompleta tipo I; e aoutra, devido a trauma. Quanto à causa da necessidade da cirurgia revisional, das oito crianças que permaneceram com implante coclear, quatro apresentaram calcificação coclear após meningite, seguida de trauma (1), malformação do nervo facial (1), falha do dispositivo interno da IC (1) e torção do feixe de eletrodos (1).

**Conclusão:**

A incidência de cirurgias revisionais foi de 4,23%. O pós-operatório das cirurgias revisionais permite uma melhoria no desempenho de audição e na linguagem dos pacientes, indicando que essas cirurgias são válidas na maioria dos casos.

## Introduction

Profound sensorineural hearing loss impairs the individual's ability to adequately communicate with the environment and can have a significant impact on the lifestyle and personality development of individuals with this deficiency.^1^

Conventional hearing aids are effective in treating most hearing impairments, but some patients cannot attain word and sentence recognition, even with a powerful hearing aid. The Cochlear Implant (CI) is the current alternative for these patients.^2^

The CI is an auditory sensory prosthesis that allows individuals with severe or profound sensorineural hearing loss to have the sensation of hearing and recognize the sounds of speech through electrical stimulation of the auditory nerve fibers. It consists of an external and an internal unit, with the latter being inserted surgically. The internal CI device implantation surgery is not completely free of risks and may present problems that will require revision surgeries.^3^, ^4^

The first report of a CI revision surgery occurred in 1985, by Hochmair-Desoyer and Burian.^5^ Since then, several reports have addressed the safety of this procedure, including the preservation or increase of speech perception performance, although there have also been reports of decreases in electrode activation, decreased speech perception and intracochlear trauma, suggesting that cochlear reimplantation may have negative functional consequences in some patients, requiring careful consideration of the expected indications and benefits.^6^, ^7^, ^8^

Indications for reimplantation follow the classification proposed by Zeitler. It includes hardware and software failure, device infection or extrusion, inadequate initial placement, surgical wound or skin flap complications, and improved cochlear implant technology. Hardware failure is defined as the complete interruption of auditory input with interrupted communication between internal and external components. It is diagnosed by a failure in the CI integrity test. Software failure is suspected in patients with progressive or intermittent performance failure or with non-auditory complaints such as earache, facial nerve stimulation, vertigo, or tinnitus. Device infection may appear as redness and loss of integrity of the skin over the receptor stimulator or an ulcerated wound. Once an infection or device exposure is suspected, antibiotics should be started immediately. If the infection persists, device explantation is recommended, and reimplantation surgery can be planned at three to four months after the event. Extrusion of electrodes accompanied by decreased auditory performance also requires reimplantation surgery. Factors related to the CI internal unit extrusion may be classified as intracochlear, such as neo-ossification that may push the bundle of electrodes out of the cochlea, or extracochlear, such as adhesions and fibrotic bands within the mastoid that may pull the electrode bundle.

Especially in children, skull growth and other extrinsic factors, such as trauma or infection, can cause migration of the CI internal unit. Currently, CI revision surgery is not indicated to update the cochlear implantation technology, but it is expected that the number of reimplantation surgeries will markedly increase in the future for this reason.^9^, ^10^

Due to the need to advise surgeons on the expected failure rate and auditory performance after reimplantation surgery, reports of revision surgeries are required. Therefore, it is highly recommended to have updated studies that can clarify these problems and to analyze whether the results vary over time, with the improvement of electrode technology and more modern surgical implantation techniques.^10^

Additionally, such reports can help to patients education who are candidates for CI surgery on the risks and possibilities of surgical reinterventions.

Therefore, the aim of this study was to identify the incidence of CI revision surgeries in a public service located in the countryside of the state of São Paulo, Brazil, accredited by the Ministry of Health to perform this type of surgery, its effectiveness and correlate them to the variables related to the development of auditory abilities and oral language.

## Methods

A retrospective study was carried out by reviewing the medical records of patients under 18 years of age with severe and profound bilateral hearing loss who underwent CI surgery, who had used the device for at least one year, and had undergone CI revision surgery between 2004 and 2015.

Data collection began after approval of the Research Ethics Committee of the institution, under number CAAE 65067317.4.00005440.

Therefore, the following data were obtained: age at the time of implantation; gender; etiology of the hearing loss; categories of auditory perception (Geers, 1984)^11^ and oral language (Bevilacqua et al., 1996)^12^ of each patient, in the pre- and post-CI phase; CI brand; need for surgical reintervention and the reason that led to the revision surgery.

The results for quantitative variables are shown as mean and standard deviation (mean ± SD) and categorical variables as frequency/percentages. The comparisons for independent samples were performed using the Mann–Whitney test and, for the dependent samples, the Wilcoxon rank-sum test. Correlations were assessed using Spearman's test and the categorical variables using the Chi-Square test. All analysis procedures were performed using JMP^®^ 10.0 software (SAS Institute Inc, Cary, NC, USA). Significance was set at *p* < 0.05.

## Results

During the study period from 2004 to 2015, 265 surgeries were performed in 236 patients who were younger than 18 years of age. Of these 265 operations, eight were sequential bilateral CI implantation (3.39%).

Of the 236 patients submitted to CI surgery, 10 patients required revision surgery (4.23%). Thirty-two surgeries were necessary in these 10 children: the first 10 surgeries were performed for device implantation, one bilateral sequential implantation and 21 (7.92%) revision surgeries (Table 1).

**Table 1:** Sample characterization by number of surgeries performed from 2004 to 2015 and by individual.

	*N*	%
*Per patient*		
Number of patients submitted to CI surgery	236	100
Number of patients submitted to Bilateral CI surgery	8	3.39
Number of patients submitted to à CI revision surgery	10	4.23
*Per surgeries*		
Total number of CI surgeries	265	100
Number of CI revision surgeries	21	7.92

CI, cochlear implant.

Of the 10 children submitted to revision surgeries, five were females and five males, with a mean age of 5.1 years at the first CI surgery. The mean age at the time of the revision surgery was 6.5 years.

Initially, in these 10 children, five surgeries were performed on the left side, four on the right side, and the sequential bilateral implantation was performed in one child.

At the time of the revision surgery, only one child required change of the reimplantation side (Subject 2) (Table 2), and this change was performed after three revision surgeries on the right side and, therefore, the fourth surgery was performed on the left side, due to trauma at the CI site, followed by exposure of the electrodes (Table 3).

**Table 2:** Data related to age, gender and implanted ear of the individuals submitted to revision surgery.

Subject	Gender	Age at CI surgery (years)	Age at revision surgery	Implanted side	Re-implanted side
1	F	5.4	5.6	L	L
2	F	3.2	7.4	R	L
3	F	3.8	7.0	R	R
4	M	4.7	8.0	L	L
5	F	8.6	9.5	R	R
6	M	3.5	4.2	L	L
7	M	1.2	1.2	L	L
8	F	6.5	6.5	R	R
9	M	1.8	3.3	R and L	R and L
10	M	12.3	12.3	L	L

CI, cochlear implant; F, female; M, male; L, left; R, right.

**Table 3:** Data from patients submitted to revision surgery.

Subject	Number of CI surgeries	Number of revision surgeries	Currently has CI	Etiology of hearing loss	Cause of revision surgery
1	2	1	Yes	Idiopathic	Twisting of the electrode
2	5	4	Yes	Idiopathic	Trauma
3	5	4	No	Genetic – cochlear malformation	Genetic – cochlear malformation
4	2	1	Yes	Idiopathic	Genetic – facial nerve malformation
5	2	1	Yes	Autoimmune	CI failure
6	5	4	No	Ototoxicity	Trauma
7	3	2	Yes	Meningitis	Post-meningitis calcification – extrusion of the electrodes
8	2	1	Yes	Meningitis	Post-meningitis calcification – false trajectory
9	4	2	Yes	Meningitis	Post-meningitis calcification – CI extrusion
10	2	1	Yes	Meningitis	Post-meningitis calcification – false trajectory

CI, Cochlear Implant.

Regarding the hearing loss etiology, of the 10 children who required revision surgery, four lost hearing due to meningitis, three are still under investigation (idiopathic), one had an autoimmune pathology, one had hearing loss due to ototoxicity, and in one hearing loss was due to cochlear malformation (incomplete cochlear partition Type I). It was necessary to remove the cochlear implant internal unit in two children, without reimplantation (Subjects 3 and 6). For Subject 3, who had cochlear malformation, the reason for the removal was repetitive meningitis, which first manifested more than 2 years after the initial CI implantation. For Subject 6, the reason was external trauma to the CI region, followed by unresolved skin infection and extrusion of the device despite four revision surgical interventions.

Regarding the reason for the revision surgery, of the eight children who were successfully reimplanted, four had cochlear calcification after meningitis (two children had extrusion of the electrodes and the CI was placed in a false trajectory in two of them), followed by trauma in one child. In another child, the facial nerve position near the cochlea caused facial contraction. The CI internal device failure occurred in one child and in another it was necessary to perform the surgical revision, due to the twisting of the electrode bundle (Table 3).

Seven subjects initially received a Cochlear^®^ CI, one child received an adapted bilateral CI, and three children received the Med-el® brand of CI. It was necessary to change the CI, maintaining the same brand, in four children (Subjects 1, 2, 7 and 9). In two of them, the CI brand (Subjects 4 and 5) was changed, so that ultimately all implants were replaced by a Cochlear^®^ device. It was elected to change from the full-band electrode to the half-band perimodiolar device due to the desire to position the electrodes close to the modiolus, in order to allow intracochlear stimulation by the electric field that was more localized than the full-band electrodes (Table 4).

**Table 4:** Characterization of devices in the phases of CI and revision surgeries, per patient (*n* = 10).

Subject	Brand of 1st CI	Changed the CI	Brand of 2nd CI	Comment
1	Cochlear^®^	Yes	Cochlear^®^	
2	Cochlear^®^	Yes	Cochlear^®^	
3	Cochlear^®^	No		CI removed
4	Med-el^®^	Yes	Cochlear^®^	
5	Med-el^®^	Yes	Cochlear^®^	
6	Cochlear^®^	No		CI removed
7	Cochlear^®^	Yes	Cochlear^®^	
8	Cochlear^®^	No		
9	Bilateral Cochlear^®^	Yes – Yes		Bilateral Cochlear^®^
10	Med-el^®^	No		

CI, cochlear implant.

Data regarding the patients’ hearing and language categories were collected in the pre- and post-CI surgery phases with an interval of at least one year after the CI revision surgery. Of the 10 children submitted to the revision surgery, eight had prelingual deafness and two had post-lingual deafness (Subjects 5 and 10) (Table 5, Table 6).

**Table 5:** Distribution of the results, related to the hearing category,^11^ in the pre-CI and post-revision surgery phases and gender, per patient (*n* = 10).

Subject	Gender	Pre-CI	Post-CI	Improvement	Comment
1	F	0	1	Y	
2	F	1	6	Y	
3	F	1	0	N	CI removed
4	M	1	1	N	
5	F	2	6	Y	
6	M	0	0	N	CI removed
7	M	1	3	Y	
8	F	1	3	Y	
9	M	0	3	Y	
10	M	2	6	Y	
	Mann–Whitney *p* = 0.83	Wilcoxon *p* = 0.0234			

CI, cochlear implant; F, female; M, male; Y, yes; N, no.

**Table 6:** Distribution of results related to language category,^12^ in the pre-CI and post-revision surgery phases and gender, per patient (*n* = 10).

Subject	Gender	Pre-CI	Post-CI	Improvement	Comment
1	F	1	1	N	
2	F	1	5	Y	
3	F	1	1	N	CI removed
4	M	1	1	N	
5	F	4	5	Y	
6	M	1	1	N	CI removed
7	M	1	2	Y	
8	F	1	2	Y	
9	M	1	3	Y	
10	M	2	5	Y	
	Mann–Whitney *p* = 0.91	Wilcoxon *p* = 0.0313			

CI, cochlear implant; F, female; M, male; Y, yes; N, no.

Of the eight children who were successfully reimplanted, only one child did not show improvement in auditory perception (Subject 4). When we compared results related to the Hearing Category^11^ in the pre-CI and post-revision surgery phases, a significant difference was observed after the revision surgery (Wilcoxon Signed Rank test, *p* = 0.0234) (Table 5).

There was no correlation between the results of the Hearing Category, obtained in the two studied phases, when considering the age variable (Spearman's rho = 0.13; *p* = 0.723); nor there was any association with gender (Mann–Whitney test, *p* = 0.83). A weak correlation was observed when analyzing the Hearing Category performance in the pre-CI and post-revision surgery phases with the subject's age at the time of the CI revision surgery (Spearman's rho = 0.31, *p* = 0.38).

The results of the subjects’ Language performance are shown in Table 6. Of the children who were successfully reimplanted, two did not show improvement in the Language Categories^12^ (Subjects 1 and 4) (Table 6).

When comparing the results related to the Language Category^12^ in the pre-CI and post-revision surgery phases, a statistically significant improvement (Wilcoxon Signed Rank Test, *p* = 0.0313) was observed in six of the subjects, with a change in at least one category (Table 6).

There was no correlation between the Language Category results in the two assessed phases by the age variable (Spearman's rho = 0.044, *p* = 0.903) and there was no association with the gender variable (Mann–Whitney test; *p* = 0.91). There was no correlation between the Language Category performance in the pre-CI and post-revision surgery phases and the subject's age at the time of the CI revision surgery (Spearman's rho = 0.203; *p* = 0.574).

## Discussion

The indication for CI internal device implantation surgery should consider the possible need for a revision operation if clinical treatment is not effective. In this study, the data showed that 4.3%, of the patients required revision surgery which is comparable to data from the literature (Lasig – 3.2%, Cote – 5.4%, Battmer – 3.8%).^13^, ^14^, ^15^ In a comparative study between adults and children, Brown et al. (2009)^16^ found a mean rate of 5.5%; 7.5% in children and 3.8% in adults. Cullen et al. (2008)^17^ found a higher percentage (11.2%) when studying two large CI services, with approximately 1000 children receiving CIs during the 14-year period.

We observed that the etiology that most frequently required revision surgery was meningitis, with four of 10 children (40%) requiring this procedure. Therefore, patients with this etiology who are candidates for CI surgery, should definitely receive information about the possible need for revision, as it is known that the chance of the need for surgical revision is higher with this pathology, due to the possibility of cochlear calcification after meningitis, which was noted in four children in this study. This incidence differs from that found by Manrique-Huarte et al. (2016)^10^ (1 of 26 patients younger than 18 years), maybe due to the higher incidence of meningitis in our region.

Cote et al. (2007)^14^ reported the need for surgical reintervention in 13.3% of the procedures performed in their service, most frequently secondary to trauma, in a population of adults and children; a traumatic etiology occurred only in children. These data corroborate the findings of our study, since in 2 (20%) of the 10 children submitted to surgical revision, trauma was the cause of the reintervention and one child had the internal device removed and not re-implanted.

Studies indicate that revision surgery in the pediatric population is more common, both because of surgical complications and the greater likelihood of trauma involving the implanted unit.^17^

Thus, we emphasis that the involved professional team the importance to advise patients and families about avoiding trauma to the implant both before CI surgery and later during their follow-up.

A genetic etiology was found in two of the 10 children (20%) who required revision surgery, corroborating the data reported by Manrique-Huarte et al. (2016),^10^ who found this etiology in 19.2% of patients younger than 18 years.

Compared with the internal device failure rates published in the literature, the present study shows a failure rate in only 10% of cases (one child), which was remedied by replacing the CI, which is different than the percentage shown by Manrique-Huarte et al. (2016),^10^ which was 65.39%, that is, 17 of 26 children and adult patients submitted to surgery at their Center. That study also reported an inadequate electrode initial placement, in 5 of 26 patients (19.2%), whereas it was 10% in the present study sample.

We observed significant improvement in hearing and language when we compared the pre- and post-CI testing in both the results of the hearing (*p* = 0.0234) and language (*p* = 0.0313) categories, similar to that presented by Rivas et al. (2008),^6^ who observed hearing performance improvement in the postoperative period in 73% of their patients. Cullen et al. (2008)^17^ considered that the children's performance after the revision surgery was probably the same or better than that before the procedure. However, this improvement may require several months or even a year after the revision surgery, which should also be part of the information provided to the family.

Another important factor to be considered is for CI providers is to analyze and record the revision surgeries regardless of the age group. The results of this analysis should support actions to improve the quality of guidelines, indications and interventions in patients who are candidates for CI surgery or CI users and, thus, reduce the incidence of reimplantation in these services.

This study suggests that, in adequately selected patients, the benefit of revision surgery may outweigh the inherent surgical risks. In the pediatric population, the parents/guardians should be advised about revision surgeries, their most frequent causes, risks and the performance of the children after the procedure.

## Conclusion

The incidence of revision surgeries was 4.23%. The postoperative period of the revision surgeries demonstrated an improvement in the subjects’ hearing and language performance, showing that the surgical indication is valid in these cases.

## Conflicts of interest

The authors declare no conflicts of interest.
